# Supplementary material for: Essential components of a definition for early antibiotic treatment failure: A scoping review
Source: PLoS One. 2023 Jun 23;18(6):e0283417. doi: 10.1371/journal.pone.0283417 (PMC10289306; doi:10.1371/journal.pone.0283417)
Supplement: S2 Table — (DOCX) [file pone.0283417.s002.docx]

PubMed search results

| Search number | Query | Filters | Results |
| --- | --- | --- | --- |
| 1 | anti-bacterial agents[Pharmacological Action] OR "anti-bacterial agents"[MeSH Terms] OR ("anti-bacterial"[All Fields] AND "agents"[All Fields]) OR "anti-bacterial agents"[All Fields] OR "antibiotic"[All Fields] OR "antibiotics"[All Fields] OR "antibiotic's"[All Fields] OR "antibiotical"[All Fields] |  | 987,596 |
| 2 | "early treatment*" OR "early therap*" OR "early antibiotic therap*" OR "early antibiotic treatment*" |  | 25,355 |
| 3 | treatment failure[MeSH Terms] OR "treatment failure"[All Fields] OR "mortality"[MeSH Terms] OR "mortality"[All Fields] OR "mortalities"[All Fields] |  | 1,472,211 |
| 4 | #1 AND #2 AND #3 |  | 510 |
| 5 | #1 AND #2 AND #3 | from 1980/1/1 - 2022/2/28 | 495 |
| 6 | "initial treatment*" OR "initial therap*" OR "initial antibiotic therap*" OR "initial antibiotic treatment*" |  | 34,721 |
| 7 | #1 AND #3 AND #6 |  | 1,163 |
| 8 | #1 AND #3 AND #6 | from 1980/1/1 - 2022/2/28 | 1,149 |
| 9 | #8 NOT #5 |  | 1,137 |

CENTRAL search results

| ID | Search | Hits |
| --- | --- | --- |
| #1 | early treatment | 4311 |
| #2 | early therapy | 202 |
| #3 | early antibiotic therapy | 26 |
| #4 | early antibiotic treatment | 36 |
| #5 | #1 OR #2 OR #3 OR #4 | 4534 |
| #6 | anti-bacterial agents | 11673 |
| #7 | antibiotic OR "antibiotics" OR "antibiotic's" OR "antibiotical" | 35586 |
| #8 | MeSH descriptor: [Anti-Bacterial Agents] explode all trees | 12855 |
| #9 | #6 OR #7 OR #8 | 41394 |
| #10 | treatment failure | 17902 |
| #11 | MeSH descriptor: [Treatment Failure] explode all trees | 3417 |
| #12 | mortality | 106745 |
| #13 | MeSH descriptor: [Mortality] explode all trees | 14030 |
| #14 | #10 OR #11 OR #12 OR #13 | 124711 |
| #15 | #5 AND #9 AND #14 | 118 |
| #16 | #15 NOT pubmed with Publication Year from 1980 to 2022, in Trials | 26 |
| #17 | initial treatment | 5549 |
| #18 | initial therapy | 1845 |
| #19 | initial antibiotic therapy | 46 |
| #20 | initial antibiotic treatment | 41 |
| #21 | #17 OR #18 OR #19 OR #20 | 7222 |
| #22 | #9 AND #14 AND #21 | 217 |
| #23 | #22 NOT #15 | 204 |
| #24 | #23 NOT pubmed with Publication Year from 1980 to 2022, in Trials | 47 |

CINAHL search results

| NO. | Search | Filter | Item |
| --- | --- | --- | --- |
| S1 | "anti-bacterial agents" or "antibiotic" or "antibiotics" or "antibiotic's" or "antibiotical" |  | 90481 |
| S2 | "early treatment" or "early therapy" or "early antibiotic therapy" or "early antibiotic treatment" |  | 11241 |
| S3 | (MH "Mortality") or (MH "Treatment Failure") or "mortality" or "mortalities" or "treatment failure" |  | 350215 |
| S4 | S1 AND S2 AND S3 | 19800101-20220231 | 116 |
| S5 | "initial treatment" or "initial therapy" or "initial antibiotic therapy" or "initial antibiotic treatment" |  | 7222 |
| S6 | S1 AND S3 AND S6 |  | 238 |
| S7 | S6 NOT S4 | 19800101-20220231 | 232 |

Web of Science search results

| 1 | Antibiotics (Title) | 80635 |
| --- | --- | --- |
| 2 | Early treatment (Topic) | 443890 |
| 3 | Treatment failure (Topic) | 252543 |
| 4 | #3 AND #2 AND #1 | 204 |
| 5 | Initial treatment (Topic) | 247537 |
| 6 | #1 AND #5 AND #3 | 215 |
