# Supplementary material for: Essential components of a definition for early antibiotic treatment failure: A scoping review
Source: PLoS One. 2023 Jun 23;18(6):e0283417. doi: 10.1371/journal.pone.0283417 (PMC10289306; doi:10.1371/journal.pone.0283417)
Supplement: S3 Table — (DOCX) [file pone.0283417.s003.docx]

**Eligible study reference list.**

| ID | References |
| --- | --- |
| 1 | Erjavec Z, De Vries-Hospers HG, Van Kamp H, Van Der Waaij D, Halie MR, Daenen SMG. Comparison of Imipenem versus Cefuroxim Plus Tobramycin as Empirical Therapy for Febrile Granulocytopenic Patients and Efficacy of Vancomycin and Aztreonam in case of Failure. Scandinavian Journal of Infectious Diseases. 1994. pp. 585–595. doi:10.3109/00365549409011817 |
| 2 | Bosi A, Laszlo D, Bacci S, Fanci R, Guidi S, Saccardi R, et al. An open evaluation of triple antibiotic therapy including vancomycin for febrile bone marrow transplant recipients with severe neutropenia. J Chemother. 1999;11: 287–292. |
| 3 | Arancibia F, Ewig S, Martinez JA, Ruiz M, Bauer T, Marcos MA, et al. Antimicrobial treatment failures in patients with community-acquired pneumonia: causes and prognostic implications. Am J Respir Crit Care Med. 2000;162: 154–160. |
| 4 | Ioanas M, Ferrer M, Cavalcanti M, Ferrer R, Ewig S, Filella X, et al. Causes and predictors of nonresponse to treatment of intensive care unit-acquired pneumonia. Crit Care Med. 2004;32: 938–945. |
| 5 | Menéndez R, Torres A, Zalacaín R, Aspa J, Martín Villasclaras JJ, Borderías L, et al. Risk factors of treatment failure in community acquired pneumonia: implications for disease outcome. Thorax. 2004;59: 960–965. |
| 6 | Edelsberg J, Berger A, Weber DJ, Mallick R, Kuznik A, Oster G. Clinical and economic consequences of failure of initial antibiotic therapy for hospitalized patients with complicated skin and skin-structure infections. Infect Control Hosp Epidemiol. 2008;29: 160–169. |
| 7 | Bruns AHW, Oosterheert JJ, Hustinx WNM, Gaillard CAJM, Hak E, Hoepelman AIM. Time for first antibiotic dose is not predictive for the early clinical failure of moderate–severe community-acquired pneumonia. Eur J Clin Microbiol Infect Dis. 2009;28: 913–919. |
| 8 | Mitja O, Pigrau C, Ruiz I, Vidal X, Almirante B, Planes A-M, et al. Predictors of mortality and impact of aminoglycosides on outcome in listeriosis in a retrospective cohort study. Journal of Antimicrobial Chemotherapy. 2009. pp. 416–423. doi:10.1093/jac/dkp180 |
| 9 | Shindo Y, Sato S, Maruyama E, Ohashi T, Ogawa M, Hashimoto N, et al. Health-care-associated pneumonia among hospitalized patients in a Japanese community hospital. Chest. 2009;135: 633–640. |
| 10 | Cheng C-Y, Sheng W-H, Wang J-T, Chen Y-C, Chang S-C. Safety and efficacy of intravenous colistin (colistin methanesulphonate) for severe multidrug-resistant Gram-negative bacterial infections. Int J Antimicrob Agents. 2010;35: 297–300. |
| 11 | Tumbarello M, Spanu T, Di Bidino R, Marchetti M, Ruggeri M, Trecarichi EM, et al. Costs of bloodstream infections caused by Escherichia coli and influence of extended-spectrum-beta-lactamase production and inadequate initial antibiotic therapy. Antimicrob Agents Chemother. 2010;54: 4085–4091. |
| 12 | Vogelaers D, De Bels D, Forêt F, Cran S, Gilbert E, Schoonheydt K, et al. Patterns of antimicrobial therapy in severe nosocomial infections: empiric choices, proportion of appropriate therapy, and adaptation rates—a multicentre, observational survey in critically ill patients. International Journal of Antimicrobial Agents. 2010. pp. 375–381. doi:10.1016/j.ijantimicag.2009.11.015 |
| 13 | Yakar T, Güçlü M, Serin E, Alişkan H, Husamettin E. A recent evaluation of empirical cephalosporin treatment and antibiotic resistance of changing bacterial profiles in spontaneous bacterial peritonitis. Dig Dis Sci. 2010;55: 1149–1154. |
| 14 | Jeon EJ, Cho S-G, Shin JW, Kim JY, Park IW, Choi BW, et al. The difference in clinical presentations between healthcare-associated and community-acquired pneumonia in university-affiliated hospital in Korea. Yonsei Med J. 2011;52: 282–287. |
| 15 | Stojadinović MM, Milovanović DR, Gajić BS. Scoring system development and validation for initial treatment failure in suppurative kidney infections. Surg Infect. 2011;12: 119–125. |
| 16 | Waltner-Toews RI, Paterson DL, Qureshi ZA, Sidjabat HE, Adams-Haduch JM, Shutt KA, et al. Clinical characteristics of bloodstream infections due to ampicillin-sulbactam-resistant, non-extended- spectrum-beta-lactamase-producing Escherichia coli and the role of TEM-1 hyperproduction. Antimicrob Agents Chemother. 2011;55: 495–501. |
| 17 | Eckburg PB, Friedland HD, Llorens L, Smith A, Witherell GW, Laudano JB, et al. Day 4 Clinical Response of Ceftaroline Fosamil Versus Ceftriaxone for Community-Acquired Bacterial Pneumonia. Infect Dis Clin Pract. 2012;20: 254. |
| 18 | Janisch T, Wendt J, Hoffmann R, Ortlepp JR. Expected and observed mortality in critically ill patients receiving initial antibiotic therapy. Wien Klin Wochenschr. 2012;124: 775–781. |
| 19 | O’Neal CS, O’Neal HR, Daniels TL, Talbot TR. Treatment outcomes in patients with third-generation cephalosporin-resistant Enterobacter bacteremia. Scandinavian Journal of Infectious Diseases. 2012. pp. 726–732. doi:10.3109/00365548.2012.681694 |
| 20 | Ott SR, Hauptmeier BM, Ernen C, Lepper PM, Nüesch E, Pletz MW, et al. Treatment failure in pneumonia: impact of antibiotic treatment and cost analysis. Eur Respir J. 2012;39: 611–618. |
| 21 | Berger A, Oster G, Edelsberg J, Huang X, Weber DJ. Initial treatment failure in patients with complicated skin and skin structure infections. Surg Infect. 2013;14: 304–312. |
| 22 | Kang CK, Kim ES, Song K-H, Kim HB, Kim TS, Kim N-H, et al. Can a routine follow-up blood culture be justified in Klebsiella pneumoniaebacteremia? a retrospective case–control study. BMC Infectious Diseases. 2013. doi:10.1186/1471-2334-13-365 |
| 23 | Maruyama T, Fujisawa T, Okuno M, Toyoshima H, Tsutsui K, Maeda H, et al. A new strategy for healthcare-associated pneumonia: a 2-year prospective multicenter cohort study using risk factors for multidrug-resistant pathogens to select initial empiric therapy. Clin Infect Dis. 2013;57: 1373–1383. |
| 24 | Robinson SB, Ernst FR, Lipkin C, Huang X. Patient Outcomes on Day 4 of Intravenous Antibiotic Therapy in Non-Intensive Care Unit Hospitalized Adults With Community-Acquired Bacterial Pneumonia. Infect Dis Clin Pract. 2014;22: 320–325. |
| 25 | Di Saverio S, Sibilio A, Giorgini E, Biscardi A, Villani S, Coccolini F, et al. The NOTA Study (Non Operative Treatment for Acute Appendicitis): prospective study on the efficacy and safety of antibiotics (amoxicillin and clavulanic acid) for treating patients with right lower quadrant abdominal pain and long-term follow-up of conservatively treated suspected appendicitis. Ann Surg. 2014;260: 109–117. |
| 26 | Wang Z, Zhang X, Wu J, Zhang W, Kuang H, Li X, et al. Diagnostic value of serum procalcitonin in identifying the etiology of non-responding community-acquired pneumonia after initial antibiotic therapy. Zhonghua Jie He He Hu Xi Za Zhi. 2014;37: 824–830. |
| 27 | Wie S-H, Kim HW, Chang U-I. Effects of gentamicin monotherapy for the initial treatment of community-onset complicated non-obstructive acute pyelonephritis due to Enterobacteriaceae in elderly and non-elderly women. Clin Microbiol Infect. 2014;20: 1211–1218. |
| 28 | Wie S-H, Ki M, Kim J, Cho YK, Lim S-K, Lee JS, et al. Clinical characteristics predicting early clinical failure after 72 h of antibiotic treatment in women with community-onset acute pyelonephritis: a prospective multicentre study. Clin Microbiol Infect. 2014;20: O721–9. |
| 29 | Chong YP, Bae I-G, Lee S-R, Chung J-W, Jun J-B, Choo EJ, et al. Clinical and economic consequences of failure of initial antibiotic therapy for patients with community-onset complicated intra-abdominal infections. PLoS One. 2015;10: e0119956. |
| 30 | Elagili F, Stocchi L, Ozuner G, Kiran RP. Antibiotics alone instead of percutaneous drainage as initial treatment of large diverticular abscess. Tech Coloproctol. 2015;19: 97–103. |
| 31 | Lodise TP, Anzueto AR, Weber DJ, Shorr AF, Yang M, Smith A, et al. Assessment of time to clinical response, a proxy for discharge readiness, among hospitalized patients with community-acquired pneumonia who received either ceftaroline fosamil or ceftriaxone in two phase III FOCUS trials. Antimicrob Agents Chemother. 2015;59: 1119–1126. |
| 32 | Torres A, Sibila O, Ferrer M, Polverino E, Menendez R, Mensa J, et al. Effect of corticosteroids on treatment failure among hospitalized patients with severe community-acquired pneumonia and high inflammatory response: a randomized clinical trial. JAMA. 2015;313: 677–686. |
| 33 | Hsieh C-C, Lee C-H, Hong M-Y, Hung Y-P, Lee N-Y, Ko W-C, et al. Propensity score-matched analysis comparing the therapeutic efficacies of cefazolin and extended-spectrum cephalosporins as appropriate empirical therapy in adults with community-onset Escherichia coli, Klebsiella spp. and Proteus mirabilis bacteraemia. Int J Antimicrob Agents. 2016;48: 712–718. |
| 34 | Jääskeläinen IH, Hagberg L, From J, Schyman T, Lehtola L, Järvinen A. Treatment of complicated skin and skin structure infections in areas with low incidence of antibiotic resistance-a retrospective population based study from Finland and Sweden. Clin Microbiol Infect. 2016;22: 383.e1–383.e10. |
| 35 | Merli M, Lucidi C, Di Gregorio V, Lattanzi B, Giannelli V, Giusto M, et al. An empirical broad spectrum antibiotic therapy in health-care-associated infections improves survival in patients with cirrhosis: A randomized trial. Hepatology. 2016;63: 1632–1639. |
| 36 | Park SY, Oh WS, Kim Y-S, Yeom JS, Choi HK, Kwak YG, et al. Health care-associated acute pyelonephritis is associated with inappropriate empiric antibiotic therapy in the ED. Am J Emerg Med. 2016;34: 1415–1420. |
| 37 | Ramirez P, Lopez-Ferraz C, Gordon M, Gimeno A, Villarreal E, Ruiz J, et al. From starting mechanical ventilation to ventilator-associated pneumonia, choosing the right moment to start antibiotic treatment. Crit Care. 2016;20: 169. |
| 38 | Babich T, Zusman O, Elbaz M, Ben-Zvi H, Paul M, Leibovici L, et al. Empirical Antibiotic Treatment Does Not Improve Outcomes in Catheter-Associated Urinary Tract Infection: Prospective Cohort Study. Clin Infect Dis. 2017;65: 1799–1805. |
| 39 | Ceccato A, Cilloniz C, Ranzani OT, Menendez R, Agusti C, Gabarrus A, et al. Treatment with macrolides and glucocorticosteroids in severe community-acquired pneumonia: A post-hoc exploratory analysis of a randomized controlled trial. PLoS One. 2017;12: e0178022. |
| 40 | Ereshefsky BJ, Al-Hasan MN, Gokun Y, Martin CA. Comparison of ß-lactam plus aminoglycoside versus ß-lactam plus fluoroquinolone empirical therapy in serious nosocomial infections due to Gram-negative bacilli. J Chemother. 2017;29: 30–37. |
| 41 | Lee C-H, Hsieh C-C, Hong M-Y, Hung Y-P, Ko W-C, Lee C-C. Comparing the therapeutic efficacies of third-generation cephalosporins and broader-spectrum β-lactams as appropriate empirical therapy in adults with community-onset monomicrobial Enterobacteriaceae bacteraemia: a propensity score matched analysis. Int J Antimicrob Agents. 2017;49: 617–623. |
| 42 | Ruiz-Ramos J, Vidal-Cortés P, Díaz-Lamas A, Reig-Valero R, Roche-Campo F, Del Valle-Ortiz M, et al. Ventilator-associated pneumonia by methicillin-susceptible Staphylococcus aureus: do minimum inhibitory concentrations to vancomycin and daptomycin matter? Eur J Clin Microbiol Infect Dis. 2017;36: 1569–1575. |
| 43 | Trupka T, Fisher K, Micek ST, Juang P, Kollef MH. Enhanced antimicrobial de-escalation for pneumonia in mechanically ventilated patients: a cross-over study. Crit Care. 2017;21: 180. |
| 44 | El-Sokkary RH, Ramadan RA, El-Shabrawy M, El-Korashi LA, Elhawary A, Embarak S, et al. Community acquired pneumonia among adult patients at an Egyptian university hospital: bacterial etiology, susceptibility profile and evaluation of the response to initial empiric antibiotic therapy. Infect Drug Resist. 2018;11: 2141–2150. |
| 45 | Karve S, Ryan K, Peeters P, Baelen E, Rojas-Farreras S, Potter D, et al. The impact of initial antibiotic treatment failure: Real-world insights in patients with complicated urinary tract infection. J Infect. 2018;76: 121–131. |
| 46 | Nie XM, Li YS, Yang ZW, Wang H, Jin SY, Jiao Y, et al. Initial empiric antibiotic therapy for community-acquired pneumonia in Chinese hospitals. Clin Microbiol Infect. 2018;24: 658.e1–658.e6. |
| 47 | Eliakim-Raz N, Babitch T, Shaw E, Addy I, Wiegand I, Vank C, et al. Risk factors for treatment failure and mortality among hospitalized patients with complicated urinary tract infection: a multicenter retrospective cohort study (RESCUING Study Group). Clin Infect Dis. 2019;68: 29–36. |
| 48 | Kim S-H, Oh S, Huh K, Cho SY, Kang C-I, Chung DR, et al. Inappropriate empirical antibiotic therapy does not adversely affect the clinical outcomes of patients with acute pyelonephritis caused by extended-spectrum β-lactamase-producing Enterobacteriales. Eur J Clin Microbiol Infect Dis. 2019;38: 937–944. |
| 49 | Peeters P, Ryan K, Karve S, Potter D, Baelen E, Rojas-Farreras S, et al. The impact of initial antibiotic treatment failure: real-world insights in patients with complicated, health care-associated intra-abdominal infection. Infect Drug Resist. 2019;12: 329–343. |
| 50 | Wongsurakiat P, Chitwarakorn N. Severe community-acquired pneumonia in general medical wards: outcomes and impact of initial antibiotic selection. BMC Pulm Med. 2019;19: 179. |
| 51 | Al-Hasan MN, Gould AP, Drennan C, Hill O, Justo JA, Kohn J, et al. Empirical fluoroquinolones versus broad-spectrum beta-lactams for Gram-negative bloodstream infections in the absence of antimicrobial resistance risk factors. J Glob Antimicrob Resist. 2020;22: 87–93. |
| 52 | Kim YJ, Lee J-M, Lee J-H. Predictive factors for early clinical response in community-onset Escherichia coli urinary tract infection and effects of initial antibiotic treatment on early clinical response. World J Clin Cases. 2020;8: 4342–4348. |
| 53 | Rac H, Gould AP, Bookstaver PB, Justo JA, Kohn J, Al-Hasan MN. Evaluation of early clinical failure criteria for gram-negative bloodstream infections. Clin Microbiol Infect. 2020;26: 73–77. |
| 54 | Shimoni Z, Salah M, Kasem A, Hermush V, Froom P. Bacterial Resistance to Cephalosporin Treatment in Elderly Stable Patients Hospitalized With a Urinary Tract Infection. Am J Med Sci. 2020;360: 243–247. |
| 55 | Herrmann L, Kimmig A, Rödel J, Hagel S, Rose N, Pletz MW, et al. Early Treatment Outcomes for Bloodstream Infections Caused by Potential AmpC Beta-Lactamase-Producing Enterobacterales with Focus on Piperacillin/Tazobactam: A Retrospective Cohort Study. Antibiotics (Basel). 2021;10. doi:10.3390/antibiotics10060665 |
| 56 | Mun SJ, Kang JS, Moon C. Procalcitonin as a predictor of early antibiotic treatment failure in patients with gram-negative bloodstream infections caused by urinary tract infections. Diagn Microbiol Infect Dis. 2021;99: 115256. |
| 57 | Garcia-Vidal C, Carratalà J. Early and late treatment failure in community-acquired pneumonia. Semin Respir Crit Care Med. 2009;30: 154–160. |
| 58 | Sánchez García M. Early antibiotic treatment failure. Int J Antimicrob Agents. 2009;34 Suppl 3: S14–9. |
| 59 | Cao B, Huang Y, She D , Cheng Q, Fan H , Tian X, er al. Diagnosis and treatment of community-acquired pneumonia in adults: 2016 clinical practice guidelines by the Chinese Thoracic Society, Chinese Medical Association. Clin Respir J. 2018;12(4):1320-1360. |
| 60 | Bassetti M, Rello J, Blasi F, Goossens H, Sotgiu G, Tavoschi L, et al. Systematic review of the impact of appropriate versus inappropriate initial antibiotic therapy on outcomes of patients with severe bacterial infections. Int J Antimicrob Agents. 2020;56: 106184. |
| 61 | Ceccato A, Torres A. Defining Clinical and Microbiological Nonresponse in Ventilator-Associated Pneumonia. Semin Respir Crit Care Med. 2022. doi:10.1055/s-0041-1740584 |
