# Supplementary material for: Essential components of a definition for early antibiotic treatment failure: A scoping review
Source: PLoS One. 2023 Jun 23;18(6):e0283417. doi: 10.1371/journal.pone.0283417 (PMC10289306; doi:10.1371/journal.pone.0283417)
Supplement: Attachment — Submitted filename: Response Letter 0306.docx [file pone.0283417.s005.docx]

Dr. Ali Amanati

Academic Editor, PLOS ONE

March 6, 2023

Dear Dr. Amanati,

On behalf of all of the authors, I would like to sincerely thank you and your team for your comments and suggestions for the improvement of our manuscript. We have incorporated all of your suggestions, as detailed below.

**Comments**

**#1**

*The manuscript's overall presentation improved after amendments and is now ‎more readable‎. I thank the authors for their very detailed ‎replies to my comments.‎*

Thank you for your support throughout this process.

*Minor correction is needed:*

*Line 267: add "(inoculum effect)" after "bacterial count". "Future studies may evaluate the importance of bacterial count (inoculum effect) and its ..."*

Response:

Thank you for your suggestion. We agree, and we have added "(inoculum effect)" after "bacterial count" in the manuscript.

Line 267

Future studies may evaluate the importance of bacterial count (inoculum effect) and its potential relevance for assessing EATF.

**#2**

*While revising your submission, please upload your figure files to the Preflight Analysis and Conversion Engine (PACE) digital diagnostic tool, https://pacev2.apexcovantage.com/.*

Response:

We appreciate your kind support. Following your instructions, we have made adjustments to "Figure 1" using the PACE tool, and re-uploaded it on the submission page.

The authors would like to thank you again for taking the time to review our manuscript. We sincerely hope that you will consider this revised draft favorably and find it acceptable for publication.

Cordially yours,

Hiroyoshi Iwata, MD, MSc, PhD, FACP

Center for Environmental and Health Sciences, Hokkaido University

Kitaku Kita 12 Nishi 7, Sapporo, 060-0812, Hokkaido, Japan

Telephone: +81-11-706-4747

E-mail1: hiwata@cehs.hokudai.ac.jp

E-mail2: hii887@mail.harvard.edu
